# Supplementary material for: Identification of Leukemia-Associated Immunophenotypes by Databaseguided Flow Cytometry Provides a Highly Sensitive and Reproducible Strategy for the Study of Measurable Residual Disease in Acute Myeloblastic Leukemia
Source: Cancers (Basel). 2022 Aug 19;14(16):4010. doi: 10.3390/cancers14164010 (PMC9406948; doi:10.3390/cancers14164010)
Supplement: Supplementary file 1 [file cancers-14-04010-s001.zip › cancers-1854485-supplementary.pdf]

Supplementary Table S1.

**Patients eligible for MRD study (CR1 after 3+7 regimen)**	**75**
**Post-remission therapy**	
Chemotherapy alone	54
ATSP	10
Allo-TMO	11
**MRD controls**	
MRD-MFC post cycle 1	75
MRD-MFC and qPCR post cycle 1	42
Sucessive MRD-MFC and qPCR monitoring	161
MRD-MFC and qPCR post cycle 2	39
MRD-MFC and qPCR post cycle 3 o +	69
**Patients non eligible for MRD study**	**70**
**Induction Chemotherapy**	
3+7 regimen (no CR)	22
ATRA based regimens (acute promyelocytic leukemia)	11
5-azacytidine based regimens	15
Other treatments	9
Palliative care	9
Early deaths	4
**Post-remission therapy**	
Chemotherapy	21
ATSP	5
Allo-TMO	6

Supplementary Table S2.

	**Specificity** mean (range)	**<0.01%**	**≥0.01-0.1%**	**>0.1**
**T1**	0,0845 (0,0002636- 1,24165)	28 (32%)	43 (49%)	16 (18%)
**T2**	0,1035 (0,0009616- 2,6067938)	17 (16%)	49 (46%)	34 (32%)
**T3**	0,0843 (0,00020497- 3,547254)	43 (42%)	43 (42%)	15 (15%)
**T4**	0,1104 (0,000247- 2,54468)	27 (30%)	45 (50%)	18 (20%)
**T5**	0,0814 (0,0002615- 2,215884)	30 (30%)	48 (48%)	21 (21%)
**T6**	0,0481 (0,00066- 0,8041249)	39 (39%)	50 (50%)	11 (11%)

Number of different LAIPs detected in the 6 first combinations of the AML/MDS panel. LAIPs were classified in 3 categories of specificity: strong, good and weak (<0.01%, ≥0.01-0.1% and >0.1). General specificity (mean + range) of LAIPs in each single combination.
*T1-6: tube 1-6
